# A Longitudinal Assessment of the Quality of Insulin Prescribing with Different Prescribing Systems

**DOI:** 10.3390/pharmacy9010053

**Published:** 2021-03-05

**Authors:** Amandeep Setra, Yogini Jani

**Affiliations:** 1 Centre for Medicines Optimisation Research and Education, University College London Hospital NHS Trust, London NW1 2BU, UK; yogini.jani@nhs.net; 2 Research Department of Practice and Policy, UCL School of Pharmacy, London WC1N 1AX, UK

**Keywords:** insulin, prescribing, medication safety, electronic prescribing, hospital, secondary care

## Abstract

Accurate and complete prescriptions of insulin are crucial to prevent medication errors from occurring. Two core components for safe insulin prescriptions are the word ‘units’ being written in full for the dose, and clear documentation of the insulin device alongside the name. A retrospective review of annual audit data was conducted for insulin prescriptions to assess the impact of changes to the prescribing system within a secondary care setting, at five time points over a period of 7 years (2014 to 2020). The review points were based on when changes were made, from standardized paper charts with a dedicated section for insulin prescribing, to a standalone hospital wide electronic prescribing and medicines administration (ePMA) system, and finally an integrated electronic health record system (EHRS). The measured outcomes were compliance with recommended standards for documentation of ‘units’ in full, and inclusion of the insulin device as part of the prescription. Overall, an improvement was seen in both outcomes of interest. Device documentation improved incrementally with each system change—34% for paper charts, 23%–56% for standalone ePMA, and 100% for ePMA integrated within EHRS. Findings highlight that differences in ePMA systems may have varying impact on safe prescribing practices.

## 1. Introduction

Insulin is a high risk medicine which is crucial for the management of diabetes in millions of patients worldwide. Although predominantly used for the treatment of type 1 diabetes, insulin is also now commonly used for the treatment of type 2 diabetes if lifestyle changes and oral medication do not help to sufficiently control glucose levels [1]. This means a growing number of patients using insulin products worldwide. The total number of insulin-requiring patients today is expected to be in excess of 200 million [2]. It is also predicted that the amount of insulin needed to effectively treat type 2 diabetes will rise by more than 20% worldwide in the next 10 years [3].

There are a number of different insulin preparations available, most of which are administered by subcutaneous injection. The three main groups of insulin available are fast-acting, intermediate-acting and long-acting. Multiple products exist within these groups. In recent years, several new insulin products have been launched, including high strength, fixed combination and biosimilar insulins [4].

The introduction of high strength insulin products to the market, combined with the increasing number of products and devices available, further increases the risk of error and consequently patient harm associated with insulin use. Multiple studies have shown that up to a quarter of insulin errors have caused patient harm [5,6], with the most common errors occurring at the prescribing and administration stage [5,7,8]. Accurate and complete prescriptions are crucial to prevent medication errors from occurring. This includes ensuring that the insulin prescription is concise, to contribute to the patient receiving the correct product at the dose intended. The 2013 UK National Diabetes inpatient audit found that prescriptions for 1.9% of patients had the word unit abbreviated to ‘u’ or written unclearly, which had the potential to cause a 10 fold overdose error if read as a ‘0’ instead of a ‘u’. This means that on any one day, 200 diabetic inpatients in England and Wales were unnecessarily exposed to the risk of a potentially catastrophic medicine error [9]. Two parameters have been highlighted as being fundamental for safe prescribing—these are that ‘units’ or ‘international units’ are written in full, and that the insulin device is clearly documented alongside the name and dose to be administered [6,10].

National, and international safety alerts [11,12,13,14,15] and studies [5,16,17,18,19,20,21] have highlighted the levels of harm that have been caused to patients from the inappropriate prescribing and administration of insulin. A multitude of errors have been reported, ranging from prescribing errors due to mis-selection from dropdown lists or wrong drug being prescribed, to administration errors due to challenges of measuring doses using syringes instead of ready to use injection devices [5,9,17,19]. Given the harm that can be caused by the inappropriate prescribing and administration of insulin, insulin is included as one of five medication related never events within the National Health Service (NHS) Improvement’s Never Event list [22]. In addition to this, insulin was included as a high risk medicine of concern by the World Health Organisation in their global challenge technical report [10]. A number of strategies have been reported for preventing errors and reducing harm from insulin prescribing and administration. These include the use of insulin syringes, education and training, and promoting self-administration of insulin within the hospital setting. [17,21,23]. Electronic prescribing systems are considered an important strategy in reducing medication errors including those involving insulin [10,24,25]. It is important to note however, that there is conflicting evidence within the literature internationally on the impact of electronic prescribing in reducing inpatient medication errors [26,27,28]. In 2019, a report from the Healthcare Safety Investigation Branch (HSIB) summarised that although the implementation of electronic prescribing is associated with a greater than 50% reduction in medication errors, different types of electronic prescribing and medicines administration (ePMA) systems are available, and this can lead to variation in how medicines are prescribed. Different ePMA systems have been shown to provide different levels of decision support leading to variation in medication error reduction. In addition to the differences in drug safety software utilised, there are also variations in how alerts are displayed [21,29]. Decision support aids safer prescribing by providing functionality such as drug dictionaries, dose suggestions and drug-drug interaction alerts.

The most recent NaDIA results (2019) for our organisation reported that 20.2% of inpatient drug charts contained one or more insulin error (prescription or glucose management). Safe use of insulin is a priority area for our organisation and has been a part of our Trust medication safety committee objectives to improve patient safety. One of the initiatives to achieve this was to improve systems for insulin prescribing.

The aim of this study was to assess the quality of insulin prescriptions at a teaching hospital in the UK, over a period of 7 years when different prescribing systems were in use. This includes both paper prescribing systems, as well as electronic prescribing systems. The objectives of the study were to review the proportion of insulin prescriptions which had clear documentation of the words ‘units’ or ‘international units’ in full, and insulin device clearly documented alongside the name and dose to be administered.

## 2. Materials and Methods

### 2.1. Setting and Study Design

This study used retrospective review of annual point prevalent audit data for insulin prescribing within a 950 bedded tertiary care, UK teaching hospital, at five time points over a period of 7 years (2014 to 2020). The review points utilised were identified to specifically focus on the years when changes were made to prescribing systems in the organisation, from paper prescription charts to electronic prescribing. Data was originally collected prospectively at each time point using prescription review on a point prevalence (data collection on single day) audit basis for all patients on insulin over two week periods. Patients were identified by a number of different methods, dependent on the system used at the time of data collection. In 2014 and 2015, patients were identified via ward pharmacists during their routine clinical screening process, in 2017 patients were identified by reviewing the electronic prescribing and medicines administration system daily, and for the 2019 and 2020 data collection periods, patients were identified using daily reports from the electronic health record system (EHRS) to identify all inpatients on a regular insulin prescription. A standardised data collection tool was used across the study periods.

The outcomes of interest were prescribing compliance with recommended standards for documentation of ‘units’ or ‘international units’ in full, and inclusion of the insulin device (vial, pen etc) as part of the prescription documentation before any annotations or changes by the pharmacist as part of their clinical review. Retrospective review of the collated data was conducted at 5 times points to assess longitudinal impact of feedback and system changes.

Patients who were identified as being on regular fixed doses of insulin were included within the analysis, if they had an active prescription. Patients who were on insulin infusions, or insulin pumps were excluded from the data analysis. All ward areas which had patients with insulin were audited during each of the data collection periods. The clinical pharmacist was aware of the audit but nurses and doctors were not aware. Ethics approval was not required for this study.

### 2.2. Prescribing Systems Used within the Study Hospital

Over the study period of 7 years, three different prescribing systems were used for the inpatient prescribing of insulin within the organization. Between 2014 to March 2015, standardized paper charts, with a dedicated area for insulin prescribing were solely used. Between March 2015 and May 2016, a dual approach was in place due to a phased implementation of a standalone electronic prescribing and administration system (ePMA) Medchart®. From May 2016 to March 2019, a standalone hospital wide electronic prescribing and administration system Medchart® was in use, and finally, from April 2019, an integrated EHRS Epic® was implemented for all inpatient areas of the organization.

For the standardised paper charts, the word ‘units’ was pre-printed on the dedicated section for prescribing insulin and the chart had a specific prompt (box) for documenting device. The functionality within both types of electronic prescribing systems ensured that the word units was always documented in full for a number of medicines, including insulin. For device documentation, the standalone ePMA system Medchart® required the prescriber to document the device within the prescription, but this was not a mandatory field. The configuration of medicines for the integrated ePMA system Epic® was different to the standalone ePMA system used from 2016–2019. The integrated system required prescription of each medicine by selecting a specific product, which must include the medicine name, formulation and device. Clinical pharmacists conducted medicines reconciliation, and reviewed prescriptions for all patients on long term insulin—this was consistent for all prescribing systems described.

## 3. Results

A total of 373 insulin prescriptions were included within the data analysis from the 5 data points. A breakdown of the number of insulin prescriptions reviewed for each time point, as well as compliance to the parameters of focus can be found in Table 1.

### 3.1. Documentation of Units/International Units in Full

All included prescriptions were analysed to ascertain whether the words units/international units were documented in full.

Over the 7 year time period the compliance with units/international units ranged from 96–100%. The results were 96% with the use of paper charts, and 100% for each of the electronic prescribing systems used as shown in Table 1.

In 2014, where paper prescription charts were in use within the organisation, the word units was written in full for insulin prescriptions for the majority of prescriptions (51/53). There were two prescriptions noted where the words units were documented as ‘u’. These prescriptions had not been prescribed using the pre-printed insulin section of the paper chart, where the word ‘units’ were pre-documented for the prescriber.

In 2015, 2017, 2019 and 2020, all prescriptions for insulin had the word units documented in full. For the 2015 data collection period, a mixture of paper and electronic prescribing systems were in use (paper 63%, ePMA 37%). Electronic prescribing systems were used within the organisation for each of the remaining time points, and the functionality within both types of electronic prescribing systems ensured that the word units was always documented in full for a number of medicines, including insulin.

### 3.2. Documentation of Device

There is a large variation noted in results for device documentation over the 5 data points (Table 1). The full implementation of ePMA in 2016 showed a reduction in device documentation, in comparison to the period when paper prescription charts were used. Paper charts within the organization had a pre-printed insulin prescription section, which had an allocated space for the prescriber to fill in the device of insulin to be used. In contrast, the standalone ePMA system required the prescriber to document the device within the prescription but this was not a mandatory field. Results whilst using the same ePMA system in 2019 yielded improved compliance with device documentation following a period of education amongst prescribers. Educational methods utilised included sessions for junior doctors to improve safety of prescribing, insulin specific teaching by the diabetes team, and inclusion of insulin safety content within organisational medication safety newsletters. With the introduction of the EHRS integrated ePMA, where medicine name and device documentation were mandatory, results improved significantly to 100% compliance.

## 4. Discussion

This longitudinal assessment has enabled us to review the impacts of three different prescribing systems on the safe prescribing of insulin within a hospital setting in a single organisation.

### 4.1. Key Findings

The study demonstrated the different impacts that different prescribing systems can have in relation to safe insulin prescribing.

Improvements were observed for the documentation of units / international units in full when the organization moved from paper prescription charts to solely using ePMA. Our findings suggest that electronic systems produced a benefit for safe and complete prescribing by ensuring that the words units are always written out in full due to the configuration of insulin prescriptions within the ePMA systems. Paper prescribing systems inherently lead to variability in prescribing as the prescriber has the freedom of where on the prescription chart the insulin is prescribed—which may in some cases mean bypassing any pre-printed insulin sections. While our findings suggest that this was not the case, it is important to note that not all hospitals who use paper prescription charts have a dedicated inpatient subcutaneous insulin chart [30].

Results were more variable for device documentation. There was a noted decrease in compliance when the organisation changed from paper prescription charts to the first ePMA system, however a significant improvement with the implementation of an ePMA integrated within the Electronic Health Record system. Documentation of device within an insulin prescription is crucial to provide clear instructions on what is to be administered to the patient [15,22]. Within the pre-printed insulin section of the paper prescribing chart, there was a dedicated section for the documentation of device type for the prescriber to complete. Documenting device within the ePMA system used between 2016–2019 was a manual process, and not a mandatory field. Improvements were noted for the documentation of device within the ePMA system between 2017 and 2019 attributed partly due to a number of other strategies implemented by the trust medication safety team, including educational interventions. These results support the utilization of education as a successful tool to improve completeness of insulin prescriptions, which is widely documented as a positive intervention [21,31,32].

Whilst evidence shows a variation amongst prescribing systems in reducing errors, our results add to research that indicates there are fewer incomplete prescriptions with electronic prescribing systems [27]. This was specifically true where mandatory fields were required prior to a prescription being accepted by the system.

### 4.2. Variations between Electronic Prescribing Systems

Our data illustrates the variation between different prescribing systems, including the configuration of medicines within these systems. This is consistent with other research that has shown that there is currently a wide variation within UK hospitals in the functionality of both electronic prescribing and paper-based systems, to enable the safe prescribing of insulin [30].

Our results support the findings by HSIB around the variability amongst ePMA systems used within the UK [33] and internationally [29,30,34]. Additionally, this study adds to international research and highlights the importance of moving from paper based systems to electronic systems, as well as the importance of how ePMA systems are or may be configured to maximize medication safety benefits [29]. With the UK moving towards using electronic prescribing for all hospital settings to help reduce medication errors, this variability is important for organisations similar to ours to consider [25].

The use of integrated electronic prescribing systems can provide additional information from previous stays and external sources, but variation still exists between different integrated systems. 

### 4.3. Interpretations and Implications for Practice

Our data shows an improvement in key prescribing parameters across different prescribing systems utilised at the same hospital, over a 7 year time frame.

Limitations of the study include differences between data collection periods, with potential inconsistencies between years, and numbers of insulin prescription analysed for each of the data collection periods. Additionally, only insulin prescriptions for regular use were included. In relation to the device documentation, the auditor did not focus on whether the device was correct based on what the patient had previously been using. It is also important to note that some of the improvements seen in the results may have come from the educational based strategies used to raise user awareness, throughout this time period. With regards to educational strategies, patients and nurses also have a role in contributing to safer prescriptions of insulin, however this was not the focus of this study.

The results with ePMA are encouraging, however there is a potential to optimize the functionality of electronic systems to improve safe prescribing of insulin in hospitals in the UK [29,30,35]. The findings provide a useful basis for further research in the field of key parameters to be included by ePMA vendors, for consistency, to ensure medication safety benefits for high risk medicines such as insulin. Incorporation of human factors is another area that requires further attention for improving ePMA to avoid errors such as selection errors within drop down menus due to sound-alike insulin names [36].

## 5. Conclusions

Implementation of electronic prescribing systems for the prescribing of insulin resulted in improved completeness for the two areas we focused on and appears to be related to the maturity and configuration of the electronic system. Our findings illustrate the importance of understanding differences between electronic prescribing systems and how these may impact prescribing practices.

## Figures and Tables

**Table 1:** Results for insulin prescribing compliance for each of the prescribing systems analysed.

	Paper Charts (August 2014)	Paper & Partial ePMA (December 2015)	ePMA (December 2017)	ePMA (January 2019)	ePMA Integrated within EHRS (February 2020)
Number of prescriptions	53	54	65	132	69
Units / international units written in full	51 (96%)	54 (100%)	65 (100%)	132 (100%)	69 (100%)
Device documented	18 (34%)	22 (41%)	15 (23%)	74 (56%)	69 (100%)

ePMA = electronic prescribing and medicines administration system; EHRS = electronic health record system.

## Data Availability

Data is contained within the article.
